# Posttranslational Arginylation Enzyme Arginyltransferase1 Shows Genetic Interactions With Specific Cellular Pathways *in vivo*

**DOI:** 10.3389/fphys.2020.00427

**Published:** 2020-05-06

**Authors:** David J. Wiley, Gennaro D’Urso, Fangliang Zhang

**Affiliations:** ^1^Department of Molecular and Cellular Pharmacology, University of Miami Leonard M. Miller School of Medicine, Miami, FL, United States; ^2^Sylvester Comprehensive Cancer Center, University of Miami Leonard M. Miller School of Medicine, Miami, FL, United States

**Keywords:** posttranslational modification, arginylation, arginyltransferase1, double-knockout screening, genetic interactions

## Abstract

Arginyltransferase1 (ATE1) is a conserved enzyme in eukaryotes mediating posttranslational arginylation, the addition of an extra arginine to an existing protein. In mammals, the dysregulations of the ATE1 gene (*ate1*) is shown to be involved in cardiovascular abnormalities, cancer, and aging-related diseases. Although biochemical evidence suggested that arginylation may be involved in stress response and/or protein degradation, the physiological role of ATE1 *in vivo* has never been systematically determined. This gap of knowledge leads to difficulties for interpreting the involvements of ATE1 in diseases pathogenesis. Since *ate1* is highly conserved between human and the unicellular organism *Schizosaccharomyces pombe* (*S. pombe*), we take advantage of the gene-knockout library of *S. pombe*, to investigate the genetic interactions between *ate1* and other genes in a systematic and unbiased manner. By this approach, we found that *ate1* has a surprisingly small and focused impact size. Among the 3659 tested genes, which covers nearly 75% of the genome of *S. pombe*, less than 5% of them displayed significant genetic interactions with *ate1*. Furthermore, these *ate1*-interacting partners can be grouped into a few discrete clustered categories based on their functions or their physical interactions. These categories include translation/transcription regulation, biosynthesis/metabolism of biomolecules (including histidine), cell morphology and cellular dynamics, response to oxidative or metabolic stress, ribosomal structure and function, and mitochondrial function. Unexpectedly, inconsistent to popular belief, very few genes in the global ubiquitination or degradation pathways showed interactions with *ate1*. Our results suggested that ATE1 specifically regulates a handful of cellular processes *in vivo*, which will provide critical mechanistic leads for studying the involvements of ATE1 in normal physiologies as well as in diseased conditions.

## Introduction

Protein posttranslational modifications (PTM) change protein properties without requiring *de novo* synthesis. Thus, PTMs are frequently relied upon to respond to acute stress or cellular signaling, and are often used to activate lateral response factors such as transcription or epigenetic modulations. For these reasons, dysregulation of PTMs are often indicated in cardiovascular diseases where stress response or cellular signaling play critical roles.

In eukaryotes, many proteins appear to be subjected to N-terminal arginylation (referred to as arginylation in this proposal), a ribosome-independent addition of one extra arginine on the N-terminus of a protein. Arginylation is catalyzed by the family of arginyltransferase (ATE). While plants contain ATE1 and ATE2, fungi and metazoans only contain ATE1, which is highly conserved across different species. Multiple lines of genetic studies have shown an important role of the ATE1 gene (*ate1*) in the cardiovascular and/or metabolic diseases in animals. For example, a genomic deletion of ATE1 was found to result in complete embryonic lethality in mice during the mid-gestation stage (E9-14) with severe defects in angiogenetic remodeling and cardiac development (Kwon et al., 2002). Moreover, organ/tissue-specific deletions of ATE1 in animals were found to cause a variety of abnormalities in morphology and function. Examples include a progressive dilated cardiomyopathy in mice when *ate1* knockout is driven by the cardiac myosin heavy chain promoter (Kurosaka et al., 2010; Kaji and Kaji, 2012; Wang et al., 2017b). Also, inducible systematic deletion of *ate1* appears to cause rapid weight loss, damaged spermatogenesis, neurological perturbations, and early lethality in adult mice (Brower and Varshavsky, 2009). In addition to these involvement in cardiovascular and metabolic abnormalities, a dysregulation of ATE1 is indicated in cancer as well. For example, reports from us and other groups showed that ATE1 is often downregulated in high grade cancer cases and is associated with poorer outcomes (Rai et al., 2015; Birnbaum et al., 2019), and that an inhibition of ATE1-mediated arginylation confers cancer cell resistance to apoptosis-induced by radiation (Masdehors et al., 2000). Furthermore, mounting evidence is also starting to indicate the involvement of ATE1 in aging-related conditions (Brower et al., 2013; Wang et al., 2017a). Unfortunately, the physiological role of ATE1 (and its arginylation activity) remains poorly understood, which adds to the difficulty of interpreting its involvements in normal conditions or diseases.

The studies about ATE1 and arginylation are still relatively scarce. Our understandings for these topics are being continuously reshaped with emerging new evidence. Arginylation has been found to take place on nearly a hundred eukaryotic proteins and the list is still expanding on a daily basis (Decca et al., 2006; Wong et al., 2007; Piatkov et al., 2012). Considering that a wide range of proteins are substrates of arginylation, it is reasonable to speculate that ATE1 may act as a root regulator of multiple cellular processes.

A popular theory about arginylation is its involvement in global protein degradation. Arginylation was shown to promote hyper-ubiquitination of the substrate proteins, which is then shown to be degraded by proteasome or autophagy (Saha and Kashina, 2011; Varshavsky, 2011). Based on mostly artificial substrates and *in vitro* data, arginylation was proposed to take place on proteins bearing certain amino acids on the 2nd residue on the N-terminus. These include the amino acids Asp, Glu, Asn, Gln (in fungi and mammals), and Cys (in mammal only) (Varshavsky, 2011). By this rule, in any given eukaryotic organism, at least 20–25% of its proteome would be estimated to be degraded by arginylation. Based on this assumption, arginylation was proposed as a central component in the generic protein degradation machinery (Varshavsky, 2011). However, the exact impact of arginylation on global homeostasis of proteins *in vivo* remain undetermined. In a more recent report, based on comparison of the sizes of protein spots on 2D-gels, the knockout of *ate1* in mouse cells appears to affect ∼20% of those spots on the steady-state levels. However, much of these effects appear to be derived from transcriptional changes. Also, proteasome inhibitors can only reverse the *ate1*-dependent reduction of less than 3% of the observed protein spots (Wong et al., 2007). Such a small impact size is inconsistent with the proposed role of arginylation as a global degradation machinery.

In addition to protein degradation, arginylation was also suggested to be involved in many other processes. These may include cellular response to various types of stressors such as those that are closely related to cardiovascular stresses. For example, the activity of arginylation in cells or animal tissues is altered during injury, high temperature, or exposures to high concentrations of oxidants or salt (Zanakis et al., 1984; Chakraborty et al., 1986; Shyne-Athwal et al., 1986, 1988; Luo et al., 1990; Jack et al., 1992; Chakraborty and Ingoglia, 1993; Xu et al., 1993; Wang and Ingoglia, 1997; Bongiovanni et al., 1999; Kumar et al., 2016). Furthermore, other lines of studies also suggested that ATE1 may regulate the dynamics of cytoskeleton (Karakozova et al., 2006; Saha et al., 2010, 2011; Kaji and Kaji, 2012).

While multiple lines of researches are starting to establish ATE1 as a root regulator for multiple processes in the cell, contradicting conclusions are very often seen among reports from different groups. While many of these discrepancies may arise from differences in test conditions, variations may also be generated because many past studies were focused on the role of individual arginylation substrates without considering the effects of arginylation on the other known or potential substrates. To further understand the function of ATE1 in normal or diseased conditions, it is desirable to investigate the physiological role of *ate1* in a systematic manner. Among the currently available tools for studying functional genomics, approaches based on a yeast gene-deletion library remain as one of the most robust and straight-forward methods (Boone et al., 2007; Giaever and Nislow, 2014). However, the role of ATE1 gene (*ate1*) has never been studied with such an unbiased manner, either as a specific query subject or as part of a comprehensive screening.

In this study, we took advantage of the *Schizosaccharomyces pombe* (*S. pombe*) single-gene knockout library. *S. pombe* is a commonly used test model for eukaryotic genes due to its similarity to metazoan organisms, while preserving its ease of genetic operation as a microbe. The meiosis and mating process of this organism also greatly facilitate the combination of different gene deletions to examine their synthetic effects, which can be quantitated by monitoring the growth rates of the resulting cells to deduce the genetic interactions between these two genes (Spirek et al., 2010; Wiley et al., 2014). The advantage of *S. pombe* as a model system is further demonstrated by the fact that nearly 70% of its gene have orthologs in the human genome, which is higher than *S. cerevisiae*, the other commonly used yeast as test model (Yanagida, 2002; Hoffman et al., 2015). The conserved genes including *ate1*, which is nearly 70% homologous in amino acid sequence in the core domain (∼200 residues) compared to its counterpart in human. However, unlike mammalian cells, *S. pombe* allows > 75% of its genome to be individually knocked out for functional tests (Spirek et al., 2010). These facts made *S. pombe* a desirable test model to determine the physiological role of *ate1 in vivo*.

In this study, we examined the effects of combining *ate1*-deletion with the deletions of other genes in *S. pombe*. The library we employed covered 3659 genes in *S. pombe*, which account for nearly 75% of the predicted open reading frames (up to 4940) in this organism (Decottignies et al., 2003). Surprisingly, only 173 of these genes, which is less than 5% of the effective library size, showed significant genetic interactions with *ate1*. Furthermore, many of these genes can be clustered into a few discrete groups in relation to cellular pathways. These include ribosomal component and translation regulation, gene transcription, oxidative stress response, cytoskeletal/structural components, mitochondrial function, and synthesis/metabolism of organic molecules and amino acids (such as histidine). Also, unexpectedly no significant interactions were observed between *ate1* and genes involved in the ubiquitin/proteome system (UPS). Our results indicate that ATE1 may specifically regulate several physiological pathways *in vivo*. Many of these interactions can be used to provide satisfactory explanations for many observed involvements of ATE1-mediated arginylation in cardiovascular/metabolic abnormalities and other diseased conditions. These novel findings will also provide clues for designing approaches intervening ATE1-related phenotypes in various diseases including cancer and metabolic dysregulations.

## Results

### The Arginyltransferase Gene *ate1* Showed Interactions With Only a Small Subset of Genes

While *ate1* is an essential gene for mammals, it can be knocked out in *S. pombe* without causing lethality. Although *S. pombe* usually exists in a haploid form and reproduces by symmetric division (fission), they can also be induced to perform mating, during which chromosome recombination proceeds in a relatively high rate. These unique properties allow the knockout of *ate1* to be easily combined with the knockout of other individual genes in the library, provided that different selection markers are being used to trace the knockout library and the query knockout (of *ate1*). Based on the growth of the products, compared to the parental strains, at least two types of interactions can be measured: (1) phenotype-enhancement, in which the crossing of the query results in a lower growth rate (also referred to as synthetic lethal); (2) phenotype-suppression, with a faster growth rate (also known as synthetic suppression).

By using the above-mentioned synthetic knockout approach, we examined the effects of combining *ate1*-knockout with 3721 individual knockouts in a *S. pombe* library, in which most of these genes were functionally annotated by either experimental evidence or prediction based on known orthologs. After excluding knockouts that lead to sterilization of the yeast, totally 3659 effective crossings were examined. Out of these crossings, we only found 173 of them resulted in significant phenotype enhancement (see Table 1A) or suppression (see Table 1B). This number is less than 5% of the library size.

**TABLE 1:** Genes whose knockout showing genetic interaction, either phenotype enhancement **(1A)** or suppression **(1B)** with *ate1* in *S. Pombe.*

**A. Gene knockouts showing phenotype-enhancing effects (synthetic lethality) with *ate1-* knockout in *S. pombe***						
**Gene**	**Systematic**			**Product**	**S. cerevisiae**	**Human**
**name**	**Gene ID**	***p*-Value**	***z*-Score**	**description**	**ortholog**	**ortholog**
igo1	SPAC10F6.16	8.1414E-07	−4.93195	Endosulfine (ENSA) serine/threonine protein kinase Igo1	IGO2, IGO1	ARPP19, ENSA
gor2	SPBC1773.17c	0.000027474	−4.19346	Glyoxylate reductase (predicted)	GOR1	GRHPR
	SPBC19G7.04	0.000041554	−4.09867	HMG box protein	FYV8	GCNA
lsd90	SPBC16E9.16c	0.000079902	−3.94469	Lsd90 protein		
puc1	SPBC19F5.01c	0.000083432	−3.93432	cyclin Puc1		
pun1	SPAC15A10.09c	0.000109954	−3.86751	SUR7 family protein Pun1 (predicted)	PUN1	
mas5	SPBC1734.11	0.00013273	−3.82134	DNAJ domain protein Mas5 (predicted)	YDJ1	DNAJA2,DNAJA4,DNAJA1
tps2	SPAC3G6.09c	0.000147704	−3.7949	Trehalose-phosphate synthase Tps2 (predicted)	TPS2	
spn1	SPAC4F10.11	0.00028086	−3.63235	Mitotic septin Spn1	CDC3	SEPTIN7,SEPTIN1,SEPTIN2, SEPTIN5,SEPTIN4
sut1	SPAC2F3.08	0.00034674	−3.57762	Plasma membrane sucrose/maltose:proton symporter Sut1		SLC45A2,SLC45A3
plc1	SPAC22F8.11	0.00046324	−3.50116	Phosphoinositide phospholipase C Plc1	PLC1	PLCB1,PLCH2,PLCH1,PLCB2, PLCB3,PLCB4,PLCD1,PLCD3, PLCD4,PLCL1,PLCL2,PLCG1 PLCG2
gid5	SPAC26H5.04	0.00068454	−3.3957	GID complex armadillo repeat subunit Gid5 (predicted)	VID28	ARMC8
pcr1	SPAC21E11.03c	0.00083756	−3.34008	Transcription factor Pcr1		
est1	SPBC2D10.13	0.00090084	−3.3198	Telomerase regulator Est1	EST1	SMG6
puf4	SPAC6G9.14	0.00091582	−3.31519	Pumilio family RNA-binding protein Puf4 (predicted)	PUF4,MPT5	
msa1	SPAC13G7.13c	0.00092698	−3.3118	RNA-binding protein Msa1	RIM4	
med1	SPAC2F7.04	0.00099746	−3.29124	Mediator complex subunit Med1	MED1	MED1
	SPBCPT2R1.01c	0.00106888	−3.27174	*S. pombe* specific DUF999 protein family 9		
coq7	SPBC337.15c	0.0010739	−3.27041	Ubiquinone biosynthesis protein Coq7	CAT5	COQ7
spn4	SPAC9G1.11c	0.00109358	−3.26527	Mitotic septin Spn4	CDC12	
Mam3	SPAP11E10.02c	0.00146666	−3.1812	Cell surface adhesion protein for conjugation Mam3		
tif51	SPAC26H5.10c	0.0016832	−3.14109	Translation elongation and termination factor eIF5A (predicted)	HYP2,ANB1	EIF5A, EIF5A2
prp17	SPBC6B1.10	0.0021378	−3.07038	Prp19 complex WD repeat protein Prp17	CDC40	CDC40
scs7	SPAC19G12.08	0.002199	−3.06194	ER sphingosine hydroxylase Scs7	SCS7	FA2H
pet1	SPAC22F8.04	0.0022328	−3.05739	Golgi phosphoenolpyruvate transmembrane transporter Pet1		SLC35C1
vas2	SPAP27G11.06c	0.002345	−3.04265	AP-1 adaptor complex sigma subunit Aps1	APS1	AP1S3,AP1S1,AP1S2
rgs1	SPAC22F3.12c	0.002421	−3.03305	Regulator of G-protein signaling Rgs1	SST2	
	SPAC186.08c	0.0038618	−2.88923	L-lactate dehydrogenase (predicted)		
ric1	SPAC1851.04c	0.0041792	−2.86431	Ypt/Rab-specific guanyl-nucleotide exchange factor (GEF) subunit Ric1	RIC1	RIC1
qcr8	SPAC1782.07	0.0043588	−2.85095	Ubiquinol-cytochrome-c reductase complex subunit 7	QCR8	UQCRQ
ubi5	SPAC589.10c	0.0051636	−2.79665	Ribosomal-ubiquitin fusion protein Ubi5 (predicted)	RPS31	RPS27A
mex67	SPBC1921.03c	0.006664	−2.71318	mRNA export receptor, Tap, nucleoporin Mex67	MEX67	NXF1, NXF3
rpl4302	SPBC83.02c	0.0073432	−2.68086	60S ribosomal protein L37a (predicted)	RPL43B, RPL43A	RPL37A
klp8	SPAC144.14	0.0073454	−2.68076	Kinesin-like protein Klp8		KIF13B,KIF13A,KIF16B, KIF14,KIF1C,KIF1A
rps1102	SPAC144.11	0.0077726	−2.66179	40S ribosomal protein S11 (predicted)	RPS11B,RPS11A	RPS11
	SPBC56F2.05c	0.007884	−2.657	Transcription factor (predicted)		
	SPAC17C9.11c	0.0080866	−2.64843	zf-C2H2 type zinc finger protein/UBA domain protein		UBXN1
ppr5	SPAC1093.01	0.0088172	−2.61906	Mitochondrial PPR repeat protein Ppr5		
rrp16	SPAC22F8.09	0.0092822	−2.60148	rRNA processing protein Rrp16 (predicted)	NOP53	NOP53
fsv1	SPAC6F12.03c	0.0098672	−2.58045	SNARE Fsv1	SYN8	STX8
	SPBC354.07c	0.010096	−2.57252	Sterol intermembrane transfer protein (predicted)	OSH7,OSH6,HES1,KES1	OSBPL9,OSBPL10,OSBPL11
	SPAC2F3.16	0.0101944	−2.56916	Ubiquitin-protein ligase E3, implicated in DNA repair (predicted)		RCHY1
msy1	SPCC1183.11	0.010212	−2.56857	MS calcium ion channel protein Msy1		
	SPAC22G7.03	0.0102284	−2.56801	Schizosaccharomyces specific protein		
alp13	SPAC23H4.12	0.0103838	−2.56278	MRG family Clr6 histone deacetylase complex subunit Alp13	EAF3	MORF4,MORF4L2, MORF4L1
cwf11	SPBC646.02	0.0109488	−2.54433	U2-type spliceosomal complex ATPase Cwf11		AQR
rpl26	SPBC29B5.03c	0.01175	−2.51956	60S ribosomal protein L26 (predicted)	RPL26B,RPL26A	RPL26,RPL26L1
rpl29	SPBC776.01	0.0128654	−2.48747	60S ribosomal protein L29	RPL29	RPL29
	SPAC144.01	0.0142774	−2.45021	Schizosaccharomyces specific protein		
rsn1	SPBC354.08c	0.0150762	−2.43054	Golgi to plasma membrane transport protein Rsn1 (predicted)	RSN1	TMEM63B,TMEM63C, TMEM63A
clr3	SPBC800.03	0.0176608	−2.37265	Histone deacetylase (class II) Clr3	HDA1	HDAC6,HDAC10
tef103	SPBC839.15c	0.0180524	−2.36454	Translation elongation factor EF-1 alpha Ef1a-c	TEF2,TEF1	EEF1A1,EEF1A2
hos2	SPAC3G9.07c	0.0188808	−2.34788	Histone deacetylase (class I) Hos2	HOS2	HDAC1,HDAC2
pab1	SPAC227.07c	0.0193724	−2.33829	Protein phosphatase PP2A regulatory subunit B-55 Pab1	CDC55	PPP2R2D,PPP2R2A,PPP2R2B, PPP2R2C
	SPCC553.12c	0.0195392	−2.33508	Transmembrane transporter (predicted)		
mug183	SPAC6G9.03c	0.0197084	−2.33185	Histone H3.3 H4 heterotetramer chaperone Rtt106-like (predicted)	RTT106	
rpl1102	SPBC17G9.10	0.020086	−2.32475	60S ribosomal protein L11 (predicted)	RPL11B,RPL11A	RPL11
puf1	SPBC56F2.08c	0.020354	−2.31974	Pumilio family RNA-binding protein Puf1 (predicted)	JSN1,PUF2	
	SPBC1703.13c	0.021252	−2.30346	Mitochondrial carrier, inorganic phosphate (predicted)	PIC2,MIR1	SLC25A3
eaf7	SPBC16A3.19	0.021428	−2.30036	Histone acetyltransferase complex subunit Eaf7	EAF7	MRGBP
gcd1	SPCC794.01c	0.023688	−2.26214	Glucose dehydrogenase Gcd1	ZWF1	H6PD
sft1	SPAC31A2.13c	0.024076	−2.25592	SNARE Sft1 (predicted)	SFT1	BET1,BET1L
	SPAC14C4.01c	0.024618	−2.24736	DUF1770 family protein		
nod1	SPAC12B10.10	0.024672	−2.24651	Medial cortical node Gef2-related protein Nod1		
mad1	SPBC3D6.04c	0.025142	−2.23922	Mitotic spindle checkpoint protein Mad1	MAD1	MAD1L1
syp1	SPBC4C3.06	0.025222	−2.238	F-BAR domain protein Syp1 (predicted)	SYP1	FCHO2,SGIP1,FCHO1
rho2	SPAC16.01	0.025592	−2.23235	Rho family GTPase Rho2	RHO2	RHOA,RHOB,RHOC
tpp1	SPAC19G12.15c	0.026072	−2.22515	Trehalose-6-phosphate phosphatase Tpp1	TPS2	
	SPBC1711.15c	0.027244	−2.208	Schizosaccharomyces pombe specific protein		
rps101	SPAC13G6.02c	0.02798	−2.19757	40S ribosomal protein S3a	RPS1A,RPS1B	RPS3A
cuf1	SPAC31A2.11c	0.028144	−2.19529	Nutritional copper sensing transcription factor Cuf1	CUP2,MAC1, HAA1	
apl6	SPAC23H3.06	0.029952	−2.17073	AP-3 adaptor complex subunit Apl6 (predicted)	APL6	AP3B1,AP3B2
jmj1	SPAC25H1.02	0.02998	−2.17036	Histone demethylase Jmj1 (predicted)		JMJD4
nrl1	SPBC20F10.05	0.032478	−2.13847	RNAi-mediated silencing protein, human NRDE2 ortholog Nrl1		NRDE2
atp3	SPBC1734.13	0.032508	−2.13812	F1-FO ATP synthase gamma subunit (predicted)	ATP3	ATP5F1C
oma1	SPAP14E8.04	0.033356	−2.12778	Metallopeptidase Oma1 (predicted)	OMA1	OMA1
ace2	SPAC6G10.12c	0.034516	−2.11399	Transcription factor Ace2	ACE2	
	SPACUNK4.13c	0.036738	−2.08865	Mitochondrial NTPase Obg family, human OLA1 ortholog, implicated in mitochondrial translation, ribosome assembly, or tRNA metabolism (predicted)	YLF2	OLA1
rtn1	SPBC31A8.01c	0.037292	−2.08255	Reticulon Rtn1	RTN2,RTN1	RTN1,RTN2,RTN3,RTN4
mpn1	SPAC23C11.10	0.037338	−2.08206	poly(U)-specific exoribonuclease, producing 3’ uridine cyclic phosphate ends Mpn1	USB1	USB1
rud3	SPBC119.12	0.03747	−2.08061	Golgi matrix protein Rud3 (predicted)	RUD3	TRIP11
laf1	SPAC14C4.12c	0.037782	−2.07722	Clr6 L associated factor 1 Laf1	FUN19, YOR338W	
mbx1	SPBC19G7.06	0.038252	−2.07215	MADS-box transcription factor Mbx1	ARG80,MCM1	MEF2A,MEF2B,MEF2C, MEF2D

**B. Gene knockouts showing phenotype-suppressing effects (synthetic rescue) with *ate1-* knockout in *S. pombe***						
cgr1	SPAC1556.05c	7.7134E-08	5.37371	Ribosome biogenesis CGR1 family (predicted)	CGR1	CCDC86
kap123	SPBC14F5.03c	3.4032E-07	5.09963	Karyopherin/importin beta family nuclear import signal receptor Kap123	KAP123	IPO4
rpl2802	SPCC5E4.07	3.7196E-07	5.08278	60S ribosomal protein L27/L28	RPL28	RPL27A
gcn1	SPAC18G6.05c	7.1728E-07	4.95663	Translation initiation regulator Gcn1	GCN1	GCN1
pap1	SPAC1783.07c	1.35728E-06	4.83118	Transcription factor Pap1/Caf3	YAP1	
	SPAC3F10.09	1.82054E-06	4.77239	1-(5-phosphoribosyl)-5-[(5-phosphoribosylamino) methylideneamino]imidazole-4-carboxamide isomerase (predicted)	HIS6	
mcl1	SPAPB1E7.02c	0.000002246	4.72993	DNA polymerase alpha accessory factor Mcl1	CTF4	WDHD1
mtq1	SPAC29B12.05c	4.4576E-06	4.58881	Mitochondrial N(5)-glutamine methyltransferase (predicted)	MTQ1	HEMK1
atg14	SPAC25A8.02	0.00002277	4.23584	Autophagy associated protein Atg14	ATG14	ATG14
byr3	SPAC13D6.02c	0.000032138	4.15776	Translational activator, zf-CCHC type zinc finger protein (predicted)	GIS2	CNBP, ZCCHC13
mms19	SPAC1071.02	0.000037518	4.12226	CIA machinery protein Mms19	MET18	MMS19
imt2	SPCC4F11.04c	0.000041484	4.09906	Mannosyltransferase Imt2	CSH1, SUR1	
atd1	SPAC9E9.09c	0.000043798	4.08647	Aldehyde dehydrogenase (predicted)	ALD5, ALD4, ALD6	ALDH1A2, ALDH1A1, ALDH2, ALDH1B1, ALDH1A3
crf1	SPAC22H10.11c	0.000048624	4.06214	Transcriptional corepressor for ribosomal proteins via TOR signaling pathway Crf1 (predicted)	CRF1, IFH1	
	SPCC61.05	0.000099978	3.89064	Schizosaccharomyces specific multicopy membrane protein family 1		
	SPAC29A4.09	0.00010776	3.87242	rRNA exonuclease Rrp17 (predicted)	RRP17	NOL12
his1	SPAC25G10.05c	0.000112986	3.86086	ATP phosphoribosyltransferase	HIS1	
brl1	SPCC1919.15	0.000122818	3.84043	Ubiquitin-protein ligase E3 Brl1	BRE1	RNF40
elf1	SPAC3C7.08c	0.000131224	3.82415	AAA family ATPase Elf1	NEW1	
met10	SPCC584.01c	0.000142772	3.80332	Sulfite reductase NADPH flavoprotein subunit (predicted)	MET10	
fil1	SPCC1393.08	0.000195024	3.72538	Transcription factor, zf-GATA type		
rps1802	SPCC1259.01c	0.000195418	3.72487	40S ribosomal protein S18 (predicted)	RPS18A,RPS18B	RPS18
atp1	SPAC14C4.14	0.000258	3.65419	F1-FO ATP synthase alpha subunit	ATP1	ATP5F1A
ctp1	SPCC338.08	0.00038412	3.55076	CtIP-related endonuclease	SAE2	RBBP8
cox19	SPCC1672.04c	0.00050878	3.47609	Mitochondrial copper chaperone for cytochrome c oxidase Cox19 (predicted)	COX19	COX19
rep2	SPBC2F12.11c	0.0007297	3.37817	MBF transcription factor activator Rep2		
rpl3602	SPBC405.07	0.00082778	3.34334	60S ribosomal protein L36	RPL36A,RPL36B	RPL36
ght8	SPCC548.06c	0.00086908	3.3298	Plasma membrane hexose:proton symporter, unknown specificity Ght8 (predicted)	HXT15,HXT7,HXT6,STL1, HXT13, MAL11,HXT4, HXT1,HXT5,HXT8,HXT9, HXT16, GAL2,HXT2,HXT14, HXT17,HXT11	
rpl35b	SPBC1921.01c	0.00110496	3.26234	60S ribosomal protein L35a (predicted)	RPL33B,RPL33A	RPL35A
rpl901	SPAC4G9.16c	0.00138818	3.1971	60S ribosomal protein L9	RPL9A,RPL9B	RPL9
rpl1801	SPBC11C11.07	0.00140148	3.19435	60S ribosomal protein L18	RPL18B,RPL18A	RPL18
rpl3702	SPCC1223.05c	0.00161788	3.15266	60S ribosomal protein L37 (predicted)	RPL37B,RPL37A	RPL37
	SPAC17G8.06c	0.00198848	3.09195	Dihydroxy-acid dehydratase (predicted)	ILV3	
his7	SPBC29A3.02c	0.0021838	3.06403	Phosphoribosyl-AMP cyclohydrolase/phosphoribosyl- ATP pyrophosphohydrolase His7	HIS4	
lys9	SPBC3B8.03	0.0023364	3.04376	Saccharopine dehydrogenase	LYS9	AASS
his5	SPBC21H7.07c	0.002481	3.02565	Imidazoleglycerol-phosphate dehydratase His5	HIS3	
rpl15	SPCC576.11	0.0028468	2.98382	60S ribosomal protein L15 (predicted)	RPL15A,RPL15B	RPL15
dhm1	SPCP1E11.10	0.0029186	2.97618	Ankyrin repeat protein, unknown biological role	YCR051W	
zfs1	SPBC1718.07c	0.0033112	2.93727	zf-CCCH tandem zinc finger protein, human Tristetraprolin homolog Zfs1, involved in mRNA catabolism	CTH1,TIS11	ZFP36L1, ZFP36L2, ZFP36
trm112	SPAC31A2.02	0.0035714	2.91373	eRF1 methyltransferase complex and tRNA (m2G10) methyltransferase complex regulatory subunit Trm112 (predicted)	TRM112	TRMT112
his2	SPBC1711.13	0.006125	2.74102	Histidinol dehydrogenase His2 (predicted)	HIS4	
hmt2	SPBC2G5.06c	0.0065568	2.71855	Sulfide-quinone oxidoreductase		SQOR
rps1201	SPCC962.04	0.0066316	2.7148	40S ribosomal protein S12 (predicted)	RPS12	RPS12
mms1	SPAC3H8.05c	0.0075438	2.67184	Cul8-RING ubiquitin ligase complex subunit Mms1 (predicted)	MMS1	0
rpl902	SPCC613.06	0.0100146	2.57533	60S ribosomal protein L9	RPL9A,RPL9B	RPL9
pnk1	SPAC23C11.04c	0.0101678	2.57007	DNA kinase/phosphatase Pnk1	TPP1	PNKP
dml1	SPAC30C2.06c	0.0109928	2.54293	Mitochondrial inheritance GTPase, tubulin-like (predicted)	DML1	MSTO1
	SPAC732.02c	0.0127802	2.48984	Fructose-2,6-bisphosphate 2-phosphatase activity (predicted)	FBP26	PFKFB1, PFKFB2, PFKFB3, PFKFB4
ser2	SPBC3H7.07c	0.0131908	2.47858	Phosphoserine phosphatase Ser2 (predicted)	SER2	PSPH
ifa38	SPAC4G9.15	0.0135804	2.46818	Ketoreductase involved in fatty acid elongation (predicted)	IFA38	HSDL1, HSD17B12, HSD17B3
rps2801	SPAC25G10.06	0.0137516	2.46369	40S ribosomal protein S28 (predicted)	RPS28B,RPS28A	RPS28
lip2	SPAC4F10.05c	0.0162584	2.40306	Mitochondrial lipoate-protein ligase Lip2	LIP2	LIPT2
clg1	SPBC1D7.03	0.0179078	2.36752	Cyclin-like protein involved in autophagy Clg1 (predicted)	CLG1	
arp5	SPBC365.10	0.018288	2.35974	Ino80 complex actin-like protein Arp5	ARP5	ACTR5
mre11	SPAC13C5.07	0.018601	2.35343	Mre11 nuclease	MRE11	MRE11
met14	SPAC1782.11	0.0203	2.32077	Adenylyl-sulfate kinase (predicted)	MET14	PAPSS1, PAPSS2
rps1502	SPAC1071.07c	0.022698	2.27847	40S ribosomal protein S15 (predicted)	RPS15	RPS15
	SPAC3C7.04	0.024088	2.25573	Transcription factor (predicted)		
rps2802	SPCC285.15c	0.026352	2.22097	40S ribosomal protein S28, Rps2802	RPS28B,RPS28A	RPS28
git1	SPBC21C3.20c	0.027796	2.20016	C2 domain protein Git1		
rpa12	SPCC1259.03	0.028074	2.19625	DNA-directed RNA polymerase complex I subunit Rpa12	RPA12	ZNRD1
cys2	SPBC106.17c	0.028446	2.19109	Homoserine O-acetyltransferase (predicted)		
	SPBC1A4.04	0.030716	2.16072	Schizosaccharomyces specific protein		
lys7	SPAC17C9.02c	0.03157	2.14981	Alpha-aminoadipate reductase phosphopantetheinyl transferase Lys7	LYS5	AASDHPPT
ade10	SPCPB16A4.03c	0.033428	2.1269	Bifunctional IMP cyclohydrolase/phosphoribosylaminoimidazole- carboxamide formyltransferase	ADE16,ADE17	ATIC
rpl3001	SPAC9G1.03c	0.036378	2.09268	60S ribosomal protein L30 (predicted)	RPL30	RPL30
	SPBC1271.14	0.037576	2.07945	Acetyl-CoA:L-glutamate N-acetyltransferase (predicted)	ARG7	
ftp105	SPAC17A5.16	0.04206	2.03293	Golgi localized protein, human HID1 ortholog 3, implicated in vesicle-mediated transport	ECM30	HID1
ppa2	SPBC16H5.07c	0.044676	2.00769	Serine/threonine protein phosphatase Ppa2	PPH21,PPH22	PPP2CA,PPP2CB
sod2	SPAC1486.01	0.044806	2.00647	Mitochondrial superoxide dismutase Sod2	SOD2	SOD2
gpd1	SPBC215.05	0.046392	1.99181	Glycerol-3-phosphate dehydrogenase Gpd1	GPD1	GPD1L,GPD1

*The genetic interaction was determined by the effects of combing two gene knockouts on the growth rate of the resulting yeast colony compared to the individual knockouts. Sexual mating of *S. pombe* was used to generate the crossings between the ate1-knockout and the other gene-knockouts in a library. While the original library contains 3721 different knockout strains, some of these deletion strains appear to be sterile and cannot be mated. Thus, the effective library size was 3659 in our tests. Four replicates were used to determine the effects on growth of the crossed, double-knockout colony compared to the parental strain with a single knockout (without *ate1* deletion). Based on the comparison, a result of slower growth is considered as phenotype-enhancement (in Table 1A). Vice versa, those that grow faster than the parental strain is considered phenotype-suppression (Table 1B). A confidence value (*p*-values) of 0.05 is the minimum for significance.*

### Genes That Genetically Interact With *ate1* Can Be Clustered by Biological Function

**FIGURE 1:**
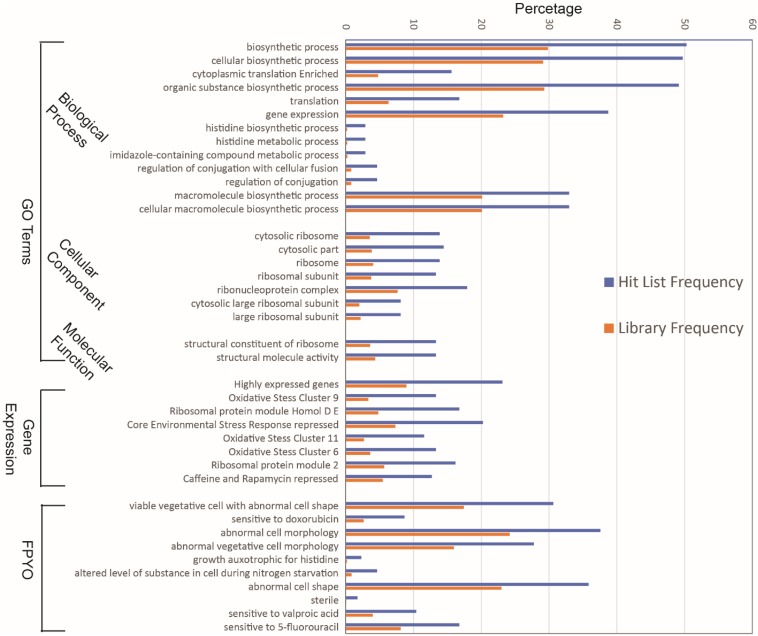
Gene features that are enriched in the *ate1*-interacting hit list compared to the library pool. The features of genes were determined by either gene ontology (GO) terms, expression category, or fission yeast phenotype ontology (FYPO).

**FIGURE 2:**
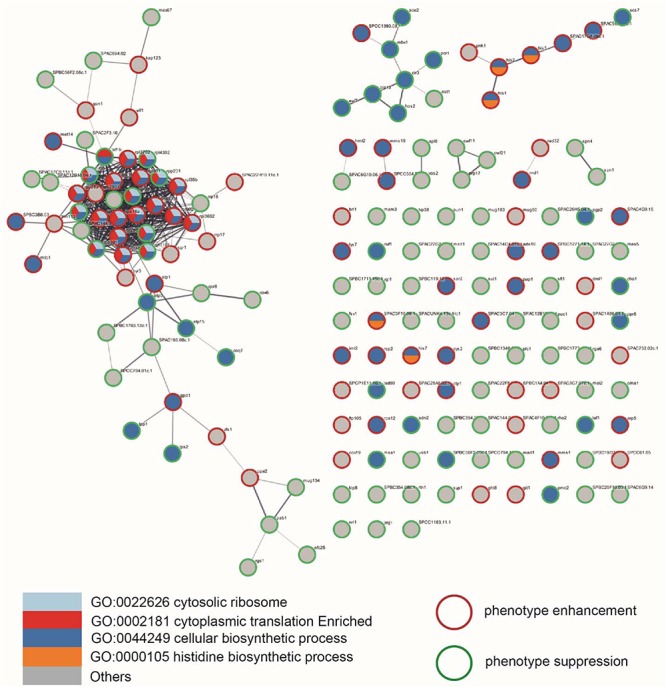
Known protein–protein interactions (PPI) between products of genes that have genetic interactions with *ate1* in *S. pombe*. The PPI database being utilized is Version 11 of STRING (https://string-db.org). The interaction map was generated with Cytoscape using the STRING App for creating the PPI information and enrichment mapping. The thickness of the connecting line represents the confidence of the experimental protein-protein interaction. A thicker line represents a higher confidence (a minimum of 0.4% confidence was used). Each rounded shape represent the product of a gene (with gene name labeled on the side). The assigned color of the rounded shape represents gene category (by GO terms), while the color of the ring (red or green) represent the direction of the genetic interaction (phenotype enhancing or suppressing).

Among the genes that showed significant interactions with *ate1*, we found specific enrichment of several types of functional relevancies. For example, simply by applying functional annotations such as gene ontology (GO), gene expression category, or Fission Yeast Phenotype Ontology (FYPO), we found that several terms are enriched or present in higher frequencies among genes interacting with *ate1* compared to the whole library (Figure 1 and Supplementary Table S1). As a further validation of the functional interactions observed between *ate1* and these genes, many of them also have known physical interactions or associations of their products among each other (Figure 2).

The enriched or increased functional annotations can be grouped into several clustered categories. Based on the frequency of presence in the hit list, the most abundant genes (nearly 50% of the hits) are those related to biosynthesis of biological molecules or proteins (Supplementary Table S1A). These include many regulators of translation or transcription (17–39%). Interestingly, among these categories, those related to histidine/amidazole synthesis/metabolism appear to be particularly impacted by *ate1*, as most of the genes associated with these pathways (5 out of 7∼9) showed interaction with *ate1* (Figure 1 and Supplementary Table S1A).

The second most abundant genre (up to 40%) is those related to cell morphology as well as cellular dynamics (fusion, conjugation, or division). These include many known regulators of cytoskeleton (Figure 1 and Supplementary Table S1B).

The third type (10∼15%) are those related to oxidative stress response, which include many highly expressed genes coding for chaperones or redox regulators (Figure 1 and Supplementary Table S1C).

The fourth are (10∼15%) ribosome components or ribosome-associated factors (Figure 1 and Supplementary Table S1A).

The fifth are those related to nutrient or metabolite stresses, such as genes related to nitrogen starvation, and genes involved in responses to metabolism interferers (caffeine, rapamycin) or metabolite analogs of lipids or nucleotides (Figure 1 and Supplementary Tables S1B,C).

In addition to the above analysis by functional annotations, the genes that have genetic interactions with *ate1* can also be grouped into a handful of clustered functional pathways or categories when being analyzed with PANTHER Classification System^1^ (Mi et al., 2013, 2019), as shown in Supplementary Table S2.

**FIGURE 3:**
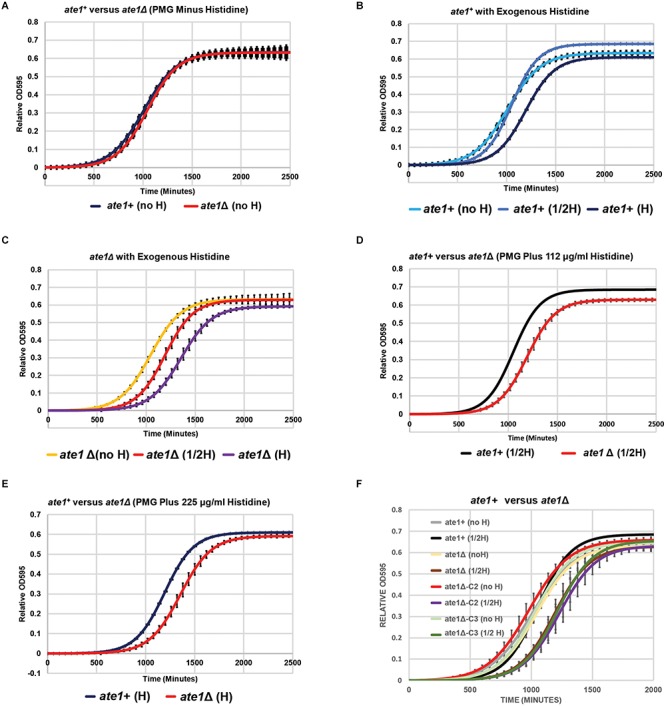
The deletion of *ate1* leads to lower tolerance of exogenous histidine in the media. The yeast strains were grown in PMG liquid media supplemented with adenine, leucine, and uracil plus histidine as indicated (no H = no histidine; 1/2H = 112 μg/ml histidine; H = 225 μg/ml histidine), while the culture density was monitor by absorbance (OD 595 nm). In the absence of histidine (referred as to “no H”), both the *ate1*Δ and the control (*ate1* +) strains grow in a similar rate (see **A**). The addition of moderate amount of histidine (112 μg/ml; “1/2H”) increases the growth of control strain (*ate1* +), while a higher concentration (225 μg/ml; “H”) exhibits cytotoxic effect as anticipated (see **B**). However, in *ate1*Δ strain, the addition of either 112 or 225 mg/ml of exogenous histidine both lead to slower growth in a dose-dependent manner (see **C**). Direct comparisons of the growth of *ate1*Δ and the control strain (*ate1* +) at different concentrations of histidine are presented at panel **(D)** and **(E)**. To exclude the possibility of clone-specific, two different clones of *ate1*Δ strain (*ate1*Δ−C2, and −C3) isolated from the same knockout process were subjected to the challenge of 112 μg/ml exogenous histidine, compared to when no histidine was added (see **F**). The curves were generated with non-linear regression. Error Bars represent standard deviation from 6 replicates.

As an example to validate the observations reached in the above non-biased study, we examined the sensitivity of *S. pombe* to exogenous histidine, which is not an essential amino acid for this organism. While a lower amount of exogenous histidine usually promotes the growth of yeasts, at high concentrations it is known to generate cytotoxicity and requires the actions of the histidine/amidazole synthesis/metabolism pathway for mitigation (Winkler and Ramos-Montanez, 2009; Watanabe et al., 2014; Duncan et al., 2018). When we compared the growth rates of the yeasts hosting the *ate1*-deletion to the control cells, we found that, although there is no apparent difference in their proliferation in the absence of exogenous histidine (Figure 3A), *ate1*-deleted yeast grow much slower than the control in high concentrations of histidine (Figures 3B–E). To ensure these observations are not specific to the colony of yeast used in the test, we repeated these experiments with two additional clones of the *ate1*-deleted *S. pombe* and reached similar conclusions (Figure 3F). Therefore, these data suggested that *ate1*-deletion indeed possesses an interaction with the histidine/amidazole synthesis/metabolism pathway.

### Many Genes Related to Mitochondria or Energy Production Genetically Interact With *ate1*

Compared to the relative abundance of literatures showing the effects of arginylation in protein degradation or cytoskeletal regulations, the potential involvements of arginylation or ATE1 in other cellular processes are less known. Interestingly, many of the observed phenotypes in animals resulted from ate1 dysregulation may be at least partly attributed to a disruption of mitochondrial function, which supplies the majority of energy molecules (ATP, NADH, and NADPH, etc.) required for many biosynthesis processes, and also constitute a major source of oxidative stressors in the cell. For example, postnatal systemic knockout of *ate1* appears to lead to drastic loss of fat and infertility, which are common consequences expected from a compromise of mitochondrial function. Consistent to this possibility, we observed a significant number (at least 19) of genes related to mitochondria or energy production showing interactions with *ate1* (Table 2). These genes represent more than 10% of the total hits, although they were not present as “enriched” because the frequency of them in the hit-list is not higher than that in the library.

**TABLE 2:** Mitochondria-related genes, as determined by GO terms, which are genetically interacting with *ate1*.

**Gene**		**# of non-sterile**	**# of genes in**	**Gene name/systematic ID**
**description**	**GO_Term**	**genes in library**	**the hits**	**in the hits list**
Mitochondrion (mitochondria)	GO:0005739	428	19	mpn1, SPACUNK4.13c, lip2, gor2, hmt2, sod2, SPAC14C4.01c, qcr8, SPAC17G8.06c, cox6, oma1, cys2, SPBC1271.14, SPBC1703.13c, atp3, atp15, coq7, rps1802, ppr5
Mitochondria inner membrane	GO:0005743	71	4	oma1, SPBC1703.13c, coq7, atp15

*The numbers of those genes in the effective library (non-sterile) and the hit list are being shown. The names (or systematic ID) of the genes in the hit list are also shown.*

### Few Genes in Global Protein Degradation Pathways Showed Genetic Interactions With *ate1*

Another surprising finding in the interacting partners of *ate1* is the rarity of genes involved in global protein ubiquitination or degradation machineries.

In principle, the uncertainties about the role of arginylation in proteome homeostasis can at least be partly addressed by the genetic interaction screening. If *ate1* is involved in global protein ubiquitination or degradation, it would be expected to have genetic interactions with the other components in these pathways. However, while many genes with known roles in global ubiquitination or degradation are present in the *S. pombe* knockout library we employed (Supplementary Table S3), only two (*ubi5* and *atg14*) showed significant interactions (either enhancing or suppressing) with *ate1* (Table 3). Among these two genes, *ubi5* is a fusion gene of ubiquitin and ribosomal component. As such, its genetic interaction with *ate1* may derive from the ribosomal component and not necessarily the ubiquitin, since no interaction was observed between *ate1* and other genes coding for ubiquitin. The rarity of interactions between *ate1* and degradation-related genes is actually consistent with the selectivity of the interacting partners of *ate1* as described above (Figure 2 and Tables 1–3). If arginylation is a generic degradation pathway for up to 20% proteins as predicted, then the expected interactions between *ate1* and other genes should be much larger than the observed number (< 5%), because many components in the pathways regulated by these arginylation-target proteins would be expected to show genetic interaction with *ate1*. Interestingly, the small impact size (< 5%) of *ate1* in genetic interactions is well consistent with the impact size (∼3%) of arginylation on the degradation of endogenous proteins as estimated in 2D-gels (Wong et al., 2007). These evidences suggest that ATE1-mediated arginylation may not be a major degradation pathway *in vivo* in the experimental condition we employed.

**TABLE 3:** Genes related to global ubiquitination and degradation, as determined by GO terms, that are in the screen library or genetically interacting with *ate1*.

		**# of genes**	
**Gene**		**in library**	**# of genes in**
**description**	**GO_term**	**(non-sterile)**	**the hits**
Protein ubiquitination	GO:0016567	19	1 (ubi5)
Ubiquitin binding	GO:0043130	25	0
Ubiquitin (protein tag)	GO:0031386	7	1 (ubi5)
Ubiquitin ligase complex	GO:0000151	20	0
Proteasome complex	GO:0000502	1	0
Lysosome	GO:0005764	5	0
Autophagy	GO:0006914	18	0
Autophagosome	GO:0005776	4	1 (atg14)
Protein catabolic process	GO:0030163	5	0

*The numbers of those genes in the effective library (non-sterile) and the hit list are being shown. The names of the genes in the hit list are also shown.*

## Discussion

Despite that *ate1* gene has been identified for 30 years, it’s *in vivo* role has remained poorly defined. For the first time, by using a systematic approach, our results showed that *ate1* possesses significant genetic interactions with a small and focused subset of genes concerning multiple critical cellular processes. The results from this study can also provide important leads for mechanistic investigations about the role of *ate1* or arginylation in normal or diseased conditions.

The power of the systematic approach employed in our study is demonstrated by the fact that many results from this unbiased investigation are highly consistent or complementing to data from past reports. For example, the observed genetic interactions between *ate1* and regulators of cell morphology are well consistent with previous reports showing the impact of arginylation on many cytoskeletal proteins (Wong et al., 2007) and cytoskeletal dynamics (Karakozova et al., 2006; Rai et al., 2008; Saha et al., 2010; Zhang et al., 2010, 2012; Kurosaka et al., 2012). The interactions between *ate1* and genes involved in oxidative stress response also support the proposed role of arginylation in stress response process (Zanakis et al., 1984; Chakraborty et al., 1986; Shyne-Athwal et al., 1986, 1988; Luo et al., 1990; Jack et al., 1992; Chakraborty and Ingoglia, 1993; Xu et al., 1993; Wang and Ingoglia, 1997; Bongiovanni et al., 1999; Kumar et al., 2016). Overall, it appears that our non-biased screening study was able to recapitulate many past observations performed in cell or animal.

Many results of this study will provide new clues for investigate the role of arginylation in physiological processes. Arginylation was shown to be involved in stress response, but the exact mechanism still awaits clarification and may benefit from the genetic interactions revealed in our study. For example, while several redox regulators, including manganese superoxide dismutase (SOD) appear to have phenotype-enhancing relationship with *ate1*, many genes related to mitochondria, the main source of reactive oxygen species (ROS) in the cell, display synthetic suppression relationship. These differences imply that ATE1 may act as a scavenger of oxidatively damaged proteins in stress response. Also, the broad interactions between *ate1* and many transcription regulators and histone modulators may help to elucidate the observed but unexplained effects of ATE1 on global transcriptional landscape (Lee et al., 2012; Eisenach et al., 2014; Deka et al., 2016). Particularly, many genes of histone modulators appear to have a phenotype-suppression relationship with *ate1*. This is highly intriguing considering that previous studies have found that histone proteins are subjected to arginylation modification, which may also affect the other PTMs on histone (Wong et al., 2007; Saha et al., 2011). The interactions between *ate1* and genes related to biomolecule synthesis/metabolism are also of high interest because emerging evidence indicated a potential involvement of ATE1 in metabolism. As an example, the enrichment of the genes in histidine synthesis/metabolism pathways in principle is consistent with one of our previous findings about arginylation of phosphoribosyl pyrophosphate synthetase (PRPPS). This is because PRPPS is responsible for production of Phosphoribosyl Diphosphate, which serves as a precursor substrate for biosynthesis of histidine (Zhang et al., 2015). Due to the conserved nature of ATE1-mediated arginylation in eukaryotes (McGary et al., 2010), many of these findings may provide mechanistic insights for the role of ATE1/arginylation in cardiovascular diseases, metabolic dysregulations, and cancer in human. These exciting possibilities will become important directions for future studies.

The lack of interactions between ate1 and genes related to global ubiquitination or protein degradation, as revealed in our study, is quite surprising. Based on the loose consensus on amino acid sequence of known substrates, arginylation was long hypothesized as a signal for the ubiquitination and degradation of at least 20–25% members of the proteome in yeast or metazoan. Such a broad range of substrates would predict a very large impact size of genetic interactions of *ate1*. However, less than 5% of the tested genes, which cover nearly 75% of the genome in *S. pombe* yeast, showed interactions with *ate1*. Consistently, we found that *ate1* has very few interactions with components of the ubiquitination and degradation pathways. While these findings appear to be at odds with popular theories, it is worthy pointing out that the exact nature of arginylation in the degradation of endogenous proteins has not been decisively determined and the current theories about arginylation were mainly built on studies with artificial substrates (Bachmair et al., 1986; Varshavsky, 2011). It is possible that the substrate preference of ATE1 is more complexed than originally expected. For example, recent evidence suggested that the efficiency of arginylation may be affected by at least 11 residues on the N-terminus of a peptide, and the substrate preference of ATE1 may also be influenced by additional *in vivo* factors (Wang et al., 2011, 2018). Also, since the majority of arginylation takes place on the N-terminus of a protein, it must compete with N-terminally acetylation *in vivo*, which is a dominant PTM in most eukaryotes (Wang et al., 2011). Furthermore, while arginylation in many cases indeed promotes ubiquitination and degradation, exceptions are also abundant on endogenous arginylated proteins (Karakozova et al., 2006; Zhang et al., 2010, 2012, 2015). Maybe not coincidentally, past experimental attempts examining the impact of arginylation on the whole proteome also revealed a relatively small size. In one of such reports, the usage of 2D gels showed that less than 3% of individual proteins appear to be affected by arginylation on proteasome-dependent degradation (Karakozova et al., 2006; Wong et al., 2007). As such, alternative interpretation for the role of arginylation in protein ubiquitination/degradation may be needed and our study may provide clues for that. It is possible that arginylation is not activated under resting state but is specifically utilized for protein degradation during certain conditions such as stress response or nutrient deprivation. This possibility is at least indirectly supported by existing evidence showing the activation of arginylation during stress response and that arginylation preferentially takes place on oxidatively damaged proteins (Zhang et al., 1998; Kumar et al., 2016). It is also supported by the extensive genetic interactions between *ate1* and genes related to oxidative and metabolic stress as revealed in our study. However, this current study was conducted in a non-stressed condition and therefore cannot directly test this possibility, which will require further investigations for validation.

The other unexpected finding is the extensive interaction between *ate1* and ribosome-related genes. While the biological meaning of this interaction is still unclear, existing literatures may provide a few clues. Particularly, the ATE1 protein was found to at least partially co-localize with ribosome during cellular fractionation (Wang et al., 2011). Also, many ribosome-associated proteins were found to be arginylated (Wong et al., 2007; Wang et al., 2011). Based on this evidence, it is likely ATE1 may regulate the function of ribosome. In addition, current evidence suggest that arginylation may take place during co-translational stage and the outcome in arginylation-mediated degradation may be dependent on the dynamic of co-translational folding (Zhang et al., 2010). As such, it is also likely that arginylation is part of the mechanism for quality check of nascent peptide synthesis. This unexpected connection between arginylation and ribosome, as well as many other newly information about arginylation uncovered in this study, constitute intriguing directions for future research endeavors.

## Conclusion

By using a systematic approach, we found that the gene for arginyltransferase1 has specific interactions with a small subset of genes in the eukaryotic genome, which fall into a few clustered functional categories. Our data suggest that ATE1 may specifically regulate a few cellular pathways *in vivo*. These results will provide novel mechanistic clues to understand the role of protein arginylation in the development of cardiovascular system and the pathogenesis of related diseases.

## Materials and Methods

### Query Yeast Strain Creation

Growth conditions and genetic manipulations of *S. pombe* were performed as previously described (Moreno et al., 1991). The query strain containing *ate1*-deletion (*ate1*Δ) was prepared by targeted deletion with a linear DNA containing a nourseothricin-resistance gene flanked with sequence derived from the *ate1* gene (systemic ID: SPAC3C7.07c) loci. The 5′-region (394 bp) flanking the resistance gene was cloned with these primers, with underline to indicate the regions matching the genome sequence while the rest primes to the resistance cassette:

5′-FWD: TAGAACTTGGTGGATGGTATCGTGG
5′-REV: GGGGATCCGTCGACCTGCAGCGTACGAACTATTGTTTGAAAATTTCCCTGTTTAAT

The 3′-region (385 bp) flanking the resistance gene was cloned with these primers:

3′-FWD: GTTTAAACGAGCTCGAATTCATCTTATATTATCTGTCTACGTGTTTTATTTGC
3′-REV: TCCTTTCTCACCTACTATGCACTGTTTTG

The knockout was performed in an *h- leu1-32 ura4-D18 Ade6-M210 S. pombe* strain (PN572) as the parental strain with published protocols (Krawchuk and Wahls, 1999). The success of knockout is confirmed with this primer specific for the *ate1* gene: TCTTTGGATTGACAAGTTGAGAGTTG.

### Yeast Synthetic Genetic Array

The *ate1*Δ strain was grown to exponential phase in liquid PMG media (Sunrise Scientific Cat. #2060) supplemented with adenine, leucine and uracil. It was then pinned to agar plates in a 384-matrix format using a robotic platform RoToR HDA (Singer Instruments). The query strain was then crossed with individual strains in the *S. pombe* haploid deletion library containing individual gene deletions marked by a kanamycin resistance gene (Bioneer, version 4.0 equivalent) utilizing a modified SGA procedure (Dixon et al., 2008). This procedure was described in detailed in our previous publication (Wiley et al., 2014). In brief, the crossing was induced on a SPAS mating media (1% glucose, 7.3mM KH_2_PO_4_, with 45mg/L supplements of adenine, histidine, leucine, uracil and lysine-HCl, and vitamin supplement for pantothenic acid, nicotinic acid, inositol, and biotin; see this website for detailed recipe: https://dornsife.usc.edu/pombenet/media/). For germination, four replicates of each crossing product were pinned to a 1536 format on selective PAU + G418 media, which is the PMG media containing adenine (225 mg/L, Sigma Cat. #A8751), leucine (225 mg/L, Sigma Cat. #L8912), uracil (225 mg/L, Sigma Cat. #U0750), and the antibiotics G418 and nourseothricin. Colony growth was monitored for 3 days utilizing a flatbed scanner, and then quantified and compared using ScreenMill according to published protocols (Dittmar et al., 2010). Double mutants with a significant growth rate difference compared to the corresponding library gene deletion alone were scored as either slowed (referred to as “phenotype-enhancement”) or accelerated (referred to as “phenotype-suppression”) growth.

### Hit Analysis and Bio-Informatics

The gene feature enrichments (over-representation) in the hit list versus the library were examined by AnGeLi^2^ (Bitton et al., 2015). The hit list was compared directly to the genes screened in our assay using the false discovery rate setting for multiple testing with a *p*-value setting of 0.01. In addition, for this enrichment we performed pairwise interaction enrichment with 1000 permutations and allowed for the *p*-value to adjust. As a negative control, we also looked for underrepresented terms and we cannot find significant underrepresented entries based on inputs, which suggesting that our analysis did not create artifacts.

The PANTHER Classification System^3^ used in this study is Version 14.1, released 2019-03-12 (Mi et al., 2019).

The image of protein-protein interaction was created using Cytoscape with the STRING App (Szklarczyk et al., 2019). The PPI database being utilized is Version 11 of STRING^4^.

## Data Availability Statement

The raw data supporting the conclusions of this article will be made available by the authors, without undue reservation, to any qualified researcher.

## Author Contributions

DW performed most of the experiments and the analysis of data. GD’U was involved in supervising the performance of the experiments. FZ conceptualized and designed the project and also supervised the analysis of the data.

## Conflict of Interest

The authors declare that the research was conducted in the absence of any commercial or financial relationships that could be construed as a potential conflict of interest.

## Footnotes

1 www.pantherdb.org

2 http://bahlerweb.cs.ucl.ac.uk/cgi-bin/GLA/GLA_input

3 www.pantherdb.org

4 https://string-db.org
