# Supplementary material for: Posttranslational Arginylation Enzyme Arginyltransferase1 Shows Genetic Interactions With Specific Cellular Pathways in vivo
Source: Front Physiol. 2020 May 6;11:427. doi: 10.3389/fphys.2020.00427 (PMC7218141; doi:10.3389/fphys.2020.00427)
Supplement: Supplementary file 1 [file Table_1.pdf]

Supplemental Materials for "Posttranslational arginylation enzyme Arginyltransferase1 shows genetic interactions with specific cellular pathways *in vivo*" by David J. Wiley, Genaro D'Urso, and Fangliang Zhang

Inventory:

Supplemental Table S1 A, B, C

Supplemental Table S2

Supplemental Table S3 A, B, C

Supplemental Table S1. Gene features that are enriched in the *ate1*-interacting hit list compared to the library pool. The features of genes were determined by either gene ontology (GO) terms, expression category, or fission yeast phenotype ontology (FYPO), as listed in sub-tables A to C, respectively. The frequency of each category in the 173 gene list showing genetic interacting with *ate1* is compared to the frequency of the same category in the screening library (effective size 3659). Statistics was performed as described in published studies(Bitton, Schubert et al. 2015). In each table, closely related categories were grouped together and separated from other groups by a blank row. Within each group, the entries are sorted by their p values.

**Suppl Table S1A. Gene features that are enriched in the *ate1*-interacting hit list compared to the library pool, as determined by gene ontology (GO) terms,**					
**GO ID**	**GeneSet Name**	**Frequency In Hit list**	**Frequency In Library**	**p-Value**	**Gene Name/Gene ID**
GO: 0009058	biosynthetic process	50.28901734 (87/173)	29.81688986 (1091/3659)	1.21E-05	rpl29, gid5, byr3, pap1, his1, adn2, ade10, clr3, med1, brl1, SPAC3F10.09, rps2802, rpl3001, SPBC56F2.05c, tps2, rps1802, gcn1, atp15, rpa12, his5, plc1, rps1201, SPBC1271.14, ifa38, atd1, mms1, mbx1, rps1502, ubi5, gpd1, arp5, rpl901, cho1, lys9, rpl35b, mas5, SPAC3C7.04, rps2801, rpl15, hmt2, SPAC17G8.06c, est1, cys2, eaf7, ctp1, met10, coq7, rpl26, his7, tpp1, rpp201, rpl1801, atp3, his2, imt2, cuf1, rps1102, laf1, ace2, tef103, lsd90, alp13, trm112, met14, rpl2802, mug183, crf1, atp1, mcl1, rpl902, scs7, rps3001, rpl1102, hos2, rpl3602, mms19, rho2, rpl3702, rps101, ser2, mtq1, lys7, fil1, rpl4302, rep2, tif51, pcr1
GO: 0044249	cellular biosynthetic process	49.71098266 (86/173)	29.13364307 (1066/3659)	1.21E-05	rpl29, gid5, byr3, pap1, his1, adn2, ade10, clr3, med1, brl1, SPAC3F10.09, rps2802, rpl3001, SPBC56F2.05c, tps2, rps1802, gcn1, atp15, rpa12, his5, rps1201, SPBC1271.14, ifa38, atd1, mms1, mbx1, rps1502, ubi5, gpd1, arp5, rpl901, cho1, lys9, rpl35b, mas5, SPAC3C7.04, rps2801, rpl15, hmt2, SPAC17G8.06c, est1, cys2, eaf7, ctp1, met10, coq7, rpl26, his7, tpp1, rpp201, rpl1801, atp3, his2, imt2, cuf1, rps1102, laf1, ace2, tef103, lsd90, alp13, trm112, met14, rpl2802, mug183, crf1, atp1, mcl1, rpl902, scs7, rps3001, rpl1102, hos2, rpl3602, mms19, rho2, rpl3702, rps101, ser2, mtq1, lys7, fil1, rpl4302, rep2, tif51, pcr1
GO: 0002181	cytoplasmic translation Enriched	15.60693641 6185 (27/173)	4.75539765 (174/3659)	2.32E-05	rpl29, byr3, rps2802, rpl3001, rps1802, rps1201, rps1502, ubi5, rpl901, rpl35b, rps2801, rpl15, rpl26, rpp201, rpl1801, rps1102, tef103, trm112, rpl2802, rpl902, rps3001, rpl1102, rpl3602, rpl3702, rps101, rpl4302, tif51
GO: 1901576	organic substance biosynthetic process	49.13294798 (85/173)	29.2976223 (1072/3659)	2.42E-05	rpl29, gid5, byr3, pap1, his1, adn2, ade10, clr3, med1, brl1, SPAC3F10.09, rps2802, rpl3001, SPBC56F2.05c, tps2, rps1802, gcn1, atp15, rpa12, his5, plc1, rps1201, SPBC1271.14, ifa38, atd1, mms1, mbx1, rps1502, ubi5, gpd1, arp5, rpl901, cho1, lys9, rpl35b, mas5, SPAC3C7.04, rps2801, rpl15, hmt2, SPAC17G8.06c, est1, cys2, eaf7, ctp1, coq7, rpl26, his7, tpp1, rpp201, rpl1801, atp3, his2, imt2, cuf1, rps1102, laf1, ace2, tef103, lsd90, alp13, trm112, rpl2802, mug183, crf1, atp1, mcl1, rpl902, scs7, rps3001, rpl1102, hos2, rpl3602, mms19, rho2, rpl3702, rps101, ser2, mtq1, lys7, fil1, rpl4302, rep2, tif51, pcr1
GO: 0006412	translation	16.76300578 (29/173)	6.313200328 (231/3659)	0.000393 912	rpl29, byr3, rps2802, rpl3001, rps1802, gcn1, rps1201, rps1502, ubi5, rpl901, rpl35b, rps2801, rpl15, rpl26, rpp201, rpl1801, rps1102, tef103, trm112, rpl2802, rpl902, rps3001, rpl1102, rpl3602, rpl3702, rps101, mtq1, rpl4302, tif51

GO: 0010467	gene expression	38.7283237 (67/173)	23.23039082 (850/3659)	0.00160701	rpl29, byr3, pap1, adn2, clr3, med1, brl1, rps2802, rpl3001, SPBC56F2.05c, rps1802, gcn1, kae1, rpa12, rps1201, prp17, mms1, mei2, mbx1, rps1502, ubi5, arp5, elf1, rpl901, rpl35b, mex67, vgl1, mas5, mpn1, SPAC3C7.04, rpl15, rps2801, rrp16, eaf7, rpl26, rpp201, rpl1801, cwf21, cuf1, rps1102, laf1, ace2, tef103, cwf11, alp13, rpl2802, trm112, mug183, crf1, mcl1, zfs1, rpl902, rps3001, rpl1102, SPAC29A4.09, hos2, cgr1, rpl3602, rpl3702, mms19, rps101, mtq1, fil1, rpl4302, rep2, tif51, pcr1
GO: 0000105	histidine biosynthetic process	2.89017341 (5/173)	0.191309101 (7/3659)	0.00220557	his1, SPAC3F10.09, his5, his7, his2
GO: 0006547	histidine metabolic process	2.89017341 (5/173)	0.218638972 (8/3659)	0.00481857	his1, SPAC3F10.09, his5, his7, his2
GO: 0052803	imidazole-containing compound metabolic process	2.89017341 (5/173)	0.245968844 (9/3659)	0.00938479	his1, SPAC3F10.09, his5, his7, his2
GO: 0031137	regulation of conjugation with cellular fusion	4.624277457 (8/173)	0.792566275 (29/3659)	0.0137569	ppa2, msa1, mei2, git1, pab1, igo1, zfs1, rgs1
GO: 0046999	regulation of conjugation	4.624277457 (8/173)	0.792566275 (29/3659)	0.0137569	ppa2, msa1, mei2, git1, pab1, igo1, zfs1, rgs1
GO: 0009059	macromolecule biosynthetic process	32.94797688 (57/173)	20.11478546 (736/3659)	0.0182045	rpl29, byr3, pap1, adn2, clr3, med1, brl1, rps2802, rpl3001, SPBC56F2.05c, rps1802, gcn1, rpa12, rps1201, mms1, mbx1, rps1502, ubi5, arp5, rpl901, rpl35b, mas5, SPAC3C7.04, rps2801, rpl15, est1, eaf7, ctp1, rpl26, rpp201, rpl1801, cuf1, rps1102, laf1, ace2, tef103, alp13, trm112, rpl2802, mug183, crf1, mcl1, rpl902, rps3001, rpl1102, hos2, rpl3602, rho2, mms19, rpl3702, rps101, mtq1, fil1, rpl4302, rep2, tif51, pcr1
GO: 0034645	cellular macromolecule biosynthetic process	32.94797688 (57/173)	20.08745559 (735/3659)	0.0182045	rpl29, byr3, pap1, adn2, clr3, med1, brl1, rps2802, rpl3001, SPBC56F2.05c, rps1802, gcn1, rpa12, rps1201, mms1, mbx1, rps1502, ubi5, arp5, rpl901, rpl35b, mas5, SPAC3C7.04, rps2801, rpl15, est1, eaf7, ctp1, rpl26, rpp201, rpl1801, cuf1, rps1102, laf1, ace2, tef103, alp13, trm112, rpl2802, mug183, crf1, mcl1, rpl902, rps3001, rpl1102, hos2, rpl3602, rho2, mms19, rpl3702, rps101, mtq1, fil1, rpl4302, rep2, tif51, pcr1
GO: 0022626	cytosolic ribosome	13.87283237 (24/173)	3.552883301 (130/3659)	1.21E-05	rpl29, rps2802, rpl3001, rps1802, rps1201, rps1502, ubi5, rpl901, rpl35b, rps2801, rpl15, rpl26, rpp201, rpl1801, rps1102, rpl2802, rpl902, rps3001, rpl1102, rpl3602, rpl3702, rps101, rpl4302, tif51
GO: 0044445	cytosolic part	14.45086705 (25/173)	3.853511888 (141/3659)	1.21E-05	rpl29, rps2802, rpl3001, rps1802, rps1201, rps1502, ubi5, rpl901, rpl35b, rps2801, rpl15, rpl26, rpp201, rpl1801, rps1102, rpl2802, rpl902, rps3001, rpl1102, rpl3602, mms19, rpl3702, rps101, rpl4302, tif51
GO: 0005840	ribosome	13.87283237 (24/173)	4.044820989 (148/3659)	4.67E-05	rpl29, rps2802, rpl3001, rps1802, rps1201, rps1502, ubi5, rpl901, rpl35b, rps2801, rpl15, rpl26, rpp201, rpl1801, rps1102, rpl2802, rpl902, rps3001, rpl1102, rpl3602, rpl3702, rps101, rpl4302, tif51
GO: 0044391	ribosomal subunit	13.29479769 (23/173)	3.771522274 (138/3659)	5.01E-05	rpl29, rps2802, rpl3001, rps1802, rps1201, rps1502, ubi5, rpl901, rpl35b, rps2801, rpl15, rpl26, rpp201, rpl1801, rps1102, rpl2802, rpl902, rps3001, rpl1102, rpl3602, rpl3702, rps101, rpl4302
GO: 0030529	ribonucleoprotein complex	17.91907514 (31/173)	7.652364034 (280/3659)	0.00242179	rpl29, pby1, rps2802, rpl3001, rps1802, rps1201, prp17, mei2, rps1502, ubi5, rpl901, rpl35b, vgl1, rps2801, rpl15, est1, rpl26, rpp201, rpl1801, cwf21, rps1102, cwf11, rpl2802, rpl902, rps3001, rpl1102, rpl3602, rpl3702, rps101, rpl4302, tif51
GO: 0022625	cytosolic large	8.092485549 (14/173)	1.995080623 (73/3659)	0.00247116	rpl29, rpl3001, rpl901, rpl35b, rpl15, rpl26, rpp201, rpl1801, rpl2802, rpl902, rpl1102, rpl3602, rpl3702, rpl4302

	ribosomal subunit				
GO: 0015934	large ribosomal subunit	8.092485549 (14/173)	2.186389724 (80/3659)	0.006492 3	rpl29, rpl3001, rpl901, rpl35b, rpl15, rpl26, rpp201, rpl1801, rpl2802, rpl902, rpl1102, rpl3602, rpl3702, rpl4302
GO: 0003735	structural constituent of ribosome	13.29479769 (23/173)	3.580213173 (131/3659)	2.42E-05	rpl29, rps2802, rpl3001, rps1802, rps1201, rps1502, ubi5, rpl901, rpl35b, rps2801, rpl15, rpl26, rpp201, rpl1801, rps1102, rpl2802, rpl902, rps3001, rpl1102, rpl3602, rpl3702, rps101, rpl4302
GO: 0005198	structural molecule activity	13.29479769 (23/173)	4.345449576 (159/3659)	0.000512 802	rpl29, rps2802, rpl3001, rps1802, rps1201, rps1502, ubi5, rpl901, rpl35b, rps2801, rpl15, rpl26, rpp201, rpl1801, rps1102, rpl2802, rpl902, rps3001, rpl1102, rpl3602, rpl3702, rps101, rpl4302

Suppl Table S1B. Gene features that are enriched in the *ate1*-interacting hit list compared to the library pool, as determined by the fission yeast phenotype ontology (FYPO)

FYPO ID	GeneSet Name	Frequency In Hit list	Frequency In Library	P-Value	Gene Name/ID
FYPO: 0002197	viable vegetative cell with abnormal cell shape	30.635838 15 (53/173)	17.409128 18 (637/3659)	0.00509 841	pap1, clr3, ric1, brl1, rpl3001, vas2, rps1802, plc1, efc25, atd1, mms1, prp17, mbx1, lip2, kap123, clg1, gpd1, arp5, elf1, rpl901, mas5, mpn1, rpl15, pnk1, puf4, nod1, ctp1, spn4, puc1, spn1, rga6, imt2, cuf1, rps1102, SPBC1711.09c, pab1, ace2, cwf11, rud3, alp13, met14, trm112, crf1, mcl1, zfs1, rpl902, mre11, cgr1, mms19, rpl3702, rep2, sft1, pcr1
FYPO: 0000005	abnormal cell morphology	37.572254 34 (65/173)	24.186936 32 (885/3659)	0.01820 45	pap1, ppa2, adn2, ade10, clr3, ric1, brl1, rpl3001, vas2, rps1802, plc1, efc25, atd1, prp17, mms1, mbx1, lip2, kap123, gpd1, clg1, arp5, elf1, rpl901, mad1, mas5, mpn1, rpl15, pnk1, git1, est1, puf4, nod1, ctp1, dml1, spn4, puc1, spn1, rga6, imt2, cuf1, rps1102, SPBC1711.09c, rtn1, pab1, ace2, tef103, cwf11, rud3, alp13, rpl2802, trm112, met14, igo1, crf1, mcl1, zfs1, rpl902, mre11, cgr1, rpl3702, mms19, rho2, rep2, sft1, pcr1
FYPO: 0001118	abnormal vegetative cell morphology	27.745664 74 (48/173)	15.960644 98 (584/3659)	0.02067 95	pap1, ppa2, adn2, ade10, brl1, efc25, atd1, mms1, prp17, mbx1, gpd1, elf1, rpl901, mad1, mas5, mpn1, pnk1, git1, est1, nod1, ctp1, dml1, spn4, puc1, spn1, rga6, imt2, cuf1, rps1102, rtn1, pab1, ace2, tef103, cwf11, alp13, trm112, rpl2802, crf1, mcl1, zfs1, mre11, cgr1, mms19, rho2, rpl3702, rep2, sft1, pcr1
FYPO: 0001126	abnormal cell shape	35.838150 29 (62/173)	22.984421 97 (841/3659)	0.02593 28	pap1, ppa2, adn2, ade10, clr3, ric1, brl1, rpl3001, vas2, rps1802, plc1, efc25, atd1, mms1, prp17, mbx1, lip2, kap123, clg1, gpd1, arp5, elf1, rpl901, mad1, mas5, mpn1, rpl15, pnk1, est1, puf4, nod1, ctp1, spn4, puc1, spn1, rga6, imt2, cuf1, rps1102, SPBC1711.09c, rtn1, pab1, ace2, cwf11, rud3, alp13, met14, trm112, rpl2802, igo1, crf1, mcl1, zfs1, rpl902, mre11, cgr1, mms19, rho2, rpl3702, sft1, rep2, pcr1
FYPO: 0000280	sterile	1.7341040 46 (3/173)	0.0819896 15 (3/3659)	0.02962 93	efc25, igo1, rgs1

FYPO: 0002068	growth auxotrophic for histidine	2.312138728 (4/173)	0.163979229 (6/3659)	0.0206795	his1, his5, his7, his2
FYPO: 0004139	altered level of substance in cell during nitrogen starvation	4.624277457 (8/173)	0.87455589 (32/3659)	0.025247	pap1, ppa2, msa1, mei2, pab1, igo1, zfs1, pcr1
FYPO: 0003559	sensitive to doxorubicin	8.670520231 (15/173)	2.623667669 (96/3659)	0.0123224	rpa12, atd1, mms1, arp5, vgl1, cox6, git1, est1, puf4, apl6, ctp1, coq7, mcl1, mre11, cox19
FYPO: 0000115	sensitive to valproic acid	10.40462428 (18/173)	3.990161246 (146/3659)	0.0380954	pap1, ric1, puf1, vas2, clg1, lys9, mpn1, rps2801, hmt2, puf4, apl6, cys2, SPBC354.07c, rud3, mcl1, scs7, mms19, fil1
FYPO: 0004325	sensitive to 5-fluorouracil	16.76300578 (29/173)	8.11697185 (297/3659)	0.0391098	pap1, ppa2, med1, ric1, brl1, rpl3001, vas2, rpa12, efc25, clg1, ftp105, vgl1, mas5, pnk1, eaf7, rpl1801, spn1, cwf21, cuf1, ace2, rud3, alp13, igo1, crf1, scs7, hos2, cox19, mms19, fil1

**Suppl Table S1C. Gene features that are enriched in the *ate1*-interacting hit list compared to the library pool, as determined by expression category**					
**Category Name**	**GeneSet Name**	**Frequency In Hit list**	**Frequency In Library**	**P-Value**	**Gene Name/ID**
Gene Expression	Highly expressed genes	23.12138728 (40/173)	8.936867997 (327/3659)	1.21E-05	rpl29, byr3, ppa2, his1, ade10, SPBC1703.13c, rps2802, rpl3001, rps1802, qcr8, ght8, rps1201, atd1, mei2, rps1502, ubi5, clg1, gpd1, rpl901, lys9, rpl35b, vgl1, mas5, rps2801, hmt2, rpl26, rpp201, rpl1801, sod2, rps1102, rtn1, tef103, rpl2802, rpl902, rps3001, rpl1102, rpl3602, rpl3702, rps101, rpl4302
Gene Expression	Oxidative Stress Cluster 9	13.29479769 (23/173)	3.334244329 (122/3659)	1.21E-05	rpl29, rps2802, rpl3001, rps1802, rps1201, rps1502, rpl901, rpl35b, rps2801, rpl15, rpl26, rpp201, rpl1801, rps1102, met14, rpl2802, rpl902, rps3001, rpl1102, rpl3602, rpl3702, rps101, rpl4302
Gene Expression	Ribosomal protein module Homol D E	16.76300578 (29/173)	4.810057393 (176/3659)	1.21E-05	rpl29, his1, ade10, rps2802, rpl3001, rps1802, rps1201, rps1502, rpl901, rpl35b, rps2801, SPAC17G8.06c, met10, rpl26, rpp201, rpl1801, his2, rps1102, met14, rpl2802, rpl902, rps3001, rpl1102, rpl3602, rpl3702, ser2, rps101, rpl4302, tif51
Gene Expression	Core Environmental Stress Response repressed	20.23121387 (35/173)	7.324405575 (268/3659)	1.91E-05	rpl29, byr3, ade10, rps2802, rpl3001, msa1, rps1802, rpa12, his5, rps1201, mei2, rps1502, kap123, rpl901, rpl35b, rpl15, rps2801, SPAC17G8.06c, rrp16, rpl26, SPAC144.01, rpp201, rpl1801, rps1102, laf1, ace2, met14, rpl2802, rpl902, rps3001, rpl1102, cgr1, rpl3602, rps101, ser2
Gene Expression	Oxidative Stess Cluster 11	11.56069364 (20/173)	2.705657283 (99/3659)	2.27E-05	rpl29, rps2802, rpl3001, rps1802, rps1502, rpl901, rpl35b, rps2801, rpl15, rpl26, rpp201, rpl1801, rps1102, met14, rpl2802, rpl902, rps3001, rpl1102, rpl3602, rps101
Gene Expression	Oxidative Stess Cluster 6	13.29479769 (23/173)	3.607543045 (132/3659)	2.42E-05	rpl29, rps2802, rpl3001, rps1802, his5, rps1201, rps1502, rpl901, rpl35b, rps2801, rpl15, SPAC17G8.06c, rpl26, rpp201, rpl1801, rps1102, met14, rpl2802, rpl902, rps3001, rpl1102, rpl3602, rps101
Gene Expression	Ribosomal protein module 2	16.1849711 (28/173)	5.684613282 (208/3659)	0.000181039	rpl29, his1, ade10, rps2802, rpl3001, rps1802, rps1201, rpl901, lys9, rpl35b, rps2801, SPAC17G8.06c, cys2, met10, rpl26, rpp201, rpl1801, rps1102, met14, rpl2802, rpl902, rps3001, rpl1102, rpl3602, rpl3702, ser2, rps101, tif51
Gene Expression	Caffeine and Rapamycin repressed	12.71676301 (22/173)	5.46597431 (200/3659)	0.0488651	rpl29, byr3, puf1, rps2802, dhm1, rpl3001, rps1802, rpa12, rps1502, kap123, rps2801, rpl15, rrp16, SPAC144.01, rpp201, rpl1801, rps1102, met14, trm112, rpl2802, rpl1102, ser2

Supplemental Table S2. The functions of genes interacting with *ate1*, based on Panther Functional Analysis by pathway, protein class, molecular function, cellular component, or biological responses.

**Pathway**	**# of Genes**
Histidine biosynthesis (P02747)	5
EGF receptor signaling pathway (P00018)	3
FGF signaling pathway (P00021)	3
ATP synthesis (P02721)	2
Angiogenesis (P00005)	2
PDGF signaling pathway (P00047)	2
Sulfate assimilation (P02778)	2
Wnt signaling pathway (P00057)	2
Valine biosynthesis (P02785)	1
Lipoate_biosynthesis (P02750)	1
Histamine H1 receptor mediated signaling pathway (P04385)	1
Integrin signalling pathway (P00034)	1
Insulin/IGF pathway-mitogen activated protein kinase kinase/MAP kinase cascade (P00032)	1
Inflammation mediated by chemokine and cytokine signaling pathway (P00031)	1
De novo purine biosynthesis (P02738)	1
p53 pathway feedback loops 2 (P04398)	1
p53 pathway by glucose deprivation (P04397)	1
Cytoskeletal regulation by Rho GTPase (P00016)	1
Thyrotropin-releasing hormone receptor signaling pathway (P04394)	1
Ras Pathway (P04393)	1
Oxytocin receptor mediated signaling pathway (P04391)	1
Isoleucine biosynthesis (P02748)	1
Serine glycine biosynthesis (P02776)	1
p53 pathway (P00059)	1
5HT2 type receptor mediated signaling pathway (P04374)	1
VEGF signaling pathway (P00056)	1
5-Hydroxytryptamine degradation (P04372)	1

**Protein Class**	**# of Genes**
nucleic acid binding (PC00171)	34
hydrolase (PC00121)	18
oxidoreductase (PC00176)	12
enzyme modulator (PC00095)	7
transporter (PC00227)	6
transcription factor (PC00218)	5

membrane traffic protein (PC00150)	4
transferase (PC00220)	4
cytoskeletal protein (PC00085)	4
lyase (PC00144)	1
transfer/carrier protein (PC00219)	1
ligase (PC00142)	1
receptor (PC00197)	1
calcium-binding protein (PC00060)	1
isomerase (PC00135)	1

**Molecular Function**	**# of Genes**
catalytic activity (GO:0003824)	46
binding (GO:0005488)	42
structural molecule activity (GO:0005198)	22
transporter activity (GO:0005215)	9
transcription regulator activity (GO:0140110)	4
molecular function regulator (GO:0098772)	3
translation regulator activity (GO:0045182)	2

**Cellular Component**	**# of Genes**
cell (GO:0005623)	71
organelle (GO:0043226)	38
protein-containing complex (GO:0032991)	15
membrane (GO:0016020)	4

**Biological Response**	**# of Genes**
metabolic process (GO:0008152)	48
cellular process (GO:0009987)	42
localization (GO:0051179)	19
biological regulation (GO:0065007)	13
cellular component organization or biogenesis (GO:0071840)	5
response to stimulus (GO:0050896)	1
multicellular organismal process (GO:0032501)	1

Supplemental Table S3. Genes that are generally related to protein ubiquitination and degradation present in the screening library. These include genes coding for: (A) ubiquitin, ubiquitin ligase, or ubiquitin-like protein; (B) proteins that interact selectively and non-covalently with ubiquitin; (C) ubiquitin or ubiquitin-like proteins; (D) components of ubiquitin ligase complex; (E) component of proteasome; (F) components and regulators of lysosome; (G) components and regulators of lysosome; (H) components and regulators of autolysosome; (I) proteases, peptidase, and protein modification enzymes that are generally involved in protein degradation. Only two of these genes, *ubi5* and *atg14*, are present in the *ate1*-interacting hit list.

**Table S3A: Genes related to "protein ubiquitination"**			
**GO:0016567**			
**Screening Library**		**Hit List**	
**Systematic_ID**	**Gene_Name**	**Systematic_ID**	**Gene_Name**
SPAC13G6.01c	rad8	SPAC589.10c	ubi5
SPBC36B7.05c	pib1		
SPAC21E11.05c	cyp8		
SPAC20H4.10	ufd2		
SPAC328.02	dbl4		
SPAC1002.14	itt1		
SPAC11G7.02	pub1		
SPAC6G10.11c	ubi3		
SPAC17G8.10c	dma1		
SPAC589.10c	ubi5		
SPAC11G7.04	ubi1		
SPAC16C9.04c	mot2		
SPAC15A10.11	ubr11		
SPAC1565.07c	knd1		
SPAC1805.15c	pub2		
SPBC19C7.02	ubr1		
SPBC337.08c	ubi4		
SPBC16E9.11c	pub3		
SPAC1805.12c	uep1		

**Table S3B: Genes related to "ubiquitin binding"**			
**GO:0043130**			
**Screening Library**		**Hit List**	
**Systematic_ID**	**Gene_Name**	**Systematic_ID**	**Gene_Name**
SPAC343.09	ubx3		
SPAC3F10.13	ucp6		
SPAC2C4.15c	ubx2		

SPAC637.10c	rpn10		
SPBC83.01	ucp8		
SPBC16E9.02c	SPBC16E9.02c		
SPBC2D10.12	rhp23		
SPBC25H2.05	egd2		
SPBC887.04c	lub1		
SPCC1235.03	cue2		
SPCC1906.02c	cue3		
SPAC16E8.01	shd1		
SPBC3B9.09	vps36		
SPBC428.08c	clr4		
SPAC1486.02c	dsc2		
SPAC23H3.08c	bub3		
SPBC9B6.03	SPBC9B6.03		
SPBC25H2.16c	gga22		
SPAC19A8.05c	sst4		
SPAC1F3.05	gga21		
SPCC16A11.16c	rpn1302		
SPBC16A3.05c	rae1		
SPCC613.12c	raf1		
SPBC342.04	rpn1301		
SPAC31A2.14	bun107		

**Table S3C: Genes related to "Ubiquitin (protein tag)"**			
**GO:0031386**			
**Screening Library**		**Hit List**	
**Systematic_ID**	**Gene_Name**	**Systematic_ID**	**Gene_Name**
SPAC1783.06c	atg12	SPAC589.10c	ubi5
SPBC337.08c	ubi4		
SPBC365.06	pmt3		
SPAC6G10.11c	ubi3		
SPAC1805.12c	uep1		
SPAC11G7.04	ubi1		
SPAC589.10c	ubi5		

Table S3D: Genes related to “Ubiquitin Ligase Complex”			
GO:0000151			
Screening Library		Hit List	
Systematic_ID	Gene_Name	Systematic_ID	Gene_Name
SPAPB1A10.14	pof15		
SPAC13D6.04c	btb3		
SPAC6F6.02c	pof5		
SPBC1271.01c	pof13		
SPBC3H7.06c	pof9		
SPBC25B2.06c	btb2		
SPBC25B2.11	pof2		
SPBC1703.06	pof10		
SPBC56F2.01	pof12		
SPCC330.11	btb1		
SPCC338.16	pof3		
SPCC1827.08c	pof7		
SPAC20H4.10	ufd2		
SPAC17G8.10c	dma1		
SPAC1002.14	itt1		
SPBC19C7.02	ubr1		
SPAC15A10.11	ubr11		
SPAC328.02	dbl4		
SPBC839.03c	dcn1		
SPAPJ691.02	SPAPJ691.02		

Table S3E: Genes related to “Proteasome Complex”			
GO:0000502			
Screening Library		Hit List	
Systematic_ID	Gene_Name	Systematic_ID	Gene_Name
SPAC3G6.02	rpn15		

Table S3F: Genes related to “lysosome”			
GO:0005764			
Screening Library		Hit List	
Systematic_ID	Gene_Name	Systematic_ID	Gene_Name
SPAPB1A10.10c	ypt71		
SPCC777.05	gtr2		
SPCC16A11.01	sfk1		
SPBC337.13c	gtr1		
SPBC405.04c	ypt7		

Table S3G: Genes related to “autophagy”			
GO:0006914			
Screening Library		Hit List	
**Systematic_ID**	**Gene_Name**	**Systematic_ID**	**Gene_Name**
SPAC1D4.03c	aut12		
SPAC1687.09	irs4		
SPAC14C4.11	vtc2		
SPBC28E12.06c	lvs1		
SPAC1783.06c	atg12		
SPAC19B12.08	atg4		
SPAC23C4.16c	atg15		
SPAC458.06	atg1803		
SPAC589.07c	atg1801		
SPAC823.16c	atg1802		
SPBC15D4.07c	atg9		
SPBC31E1.01c	atg2		
SPBC3B9.06c	atg3		
SPBC4B4.10c	atg5		
SPBC6B1.05c	atg7		
SPBC1711.11	atg2402		
SPAC6F6.12	atg24		
SPAC227.04	atg10		

Table S3H: Genes related to “autophagosome”			
GO:0005776			
Screening Library		Hit List	
**Systematic_ID**	**Gene_Name**	**Systematic_ID**	**Gene_Name**
SPAC6F6.12	atg24	SPAC25A8.02	atg14
SPBC1711.11	atg2402		
SPBC15D4.07c	atg9		
SPAC25A8.02	atg14		

Table S3-I: Genes related to “protein catabolic process”			
GO:0030163			
Screening Library		Hit List	
Systematic_ID	Gene_Name	Systematic_ID	Gene_Name
SPAC13A11.05	ysp2		
SPBC8D2.01	gsk31		
SPCC1322.05c	lap2		
SPCC1795.09	yps1		
SPBC23E6.03c	nta1		
